# Honeybees Produce Millimolar Concentrations of Non-Neuronal Acetylcholine for Breeding: Possible Adverse Effects of Neonicotinoids

**DOI:** 10.1371/journal.pone.0156886

**Published:** 2016-06-10

**Authors:** Ignaz Wessler, Hedwig-Annabel Gärtner, Rosmarie Michel-Schmidt, Christoph Brochhausen, Luise Schmitz, Laura Anspach, Bernd Grünewald, Charles James Kirkpatrick

**Affiliations:** 1 Institute of Pathology, University Medical Center, Johannes Gutenberg University Mainz, Langenbeckstr. 1, D-55101 Mainz, Germany; 2 Institut für Bienenkunde, Polytechnische Gesellschaft Frankfurt am Main, Fachbereich Biowissenschaften, Goethe Universität Frankfurt am Main, Karl-von-Frisch-Weg 2, D-61440 Oberursel, Germany; 3 Institute of Pathology, University Regensburg, Franz-Josef-Strauss Allee 11, D- 93053 Regensburg, Germany; Weizmann Institute of Science, ISRAEL

## Abstract

The worldwide use of neonicotinoid pesticides has caused concern on account of their involvement in the decline of bee populations, which are key pollinators in most ecosystems. Here we describe a role of non-neuronal acetylcholine (ACh) for breeding of *Apis mellifera carnica* and a so far unknown effect of neonicotinoids on non-target insects. Royal jelly or larval food are produced by the hypopharyngeal gland of nursing bees and contain unusually high ACh concentrations (4–8 mM). ACh is extremely well conserved in royal jelly or brood food because of the acidic pH of 4.0. This condition protects ACh from degradation thus ensuring delivery of intact ACh to larvae. Raising the pH to ≥5.5 and applying cholinesterase reduced the content of ACh substantially (by 75–90%) in larval food. When this manipulated brood was tested in artificial larval breeding experiments, the survival rate was higher with food supplemented by 100% with ACh (6 mM) than with food not supplemented with ACh. ACh release from the hypopharyngeal gland and its content in brood food declined by 80%, when honeybee colonies were exposed for 4 weeks to high concentrations of the neonicotinoids clothianidin (100 parts per billion [ppb]) or thiacloprid (8,800 ppb). Under these conditions the secretory cells of the gland were markedly damaged and brood development was severely compromised. Even field-relevant low concentrations of thiacloprid (200 ppb) or clothianidin (1 and 10 ppb) reduced ACh level in the brood food and showed initial adverse effects on brood development. Our findings indicate a hitherto unknown target of neonicotinoids to induce adverse effects on non-neuronal ACh which should be considered when re-assessing the environmental risks of these compounds. To our knowledge this is a new biological mechanism, and we suggest that, in addition to their well documented neurotoxic effects, neonicotinoids may contribute to honeybee colony losses consecutive to a reduction of the ACh content in the brood food.

## Introduction

As early as 1914, Ewins provided the first evidence for non-neuronal ACh synthesized in ergot fungi [1], but in the following decades the biological role of ACh was predominantly focussed on its action as a neurotransmitter operating within the nervous system. During the past two decades our knowledge about the expression and functions of ACh outside the nervous system has been markedly extended. The terms non-neuronal ACh and non-neuronal cholinergic system describe the expression in almost all taxa, thus indicating an important role from the beginning of life, that is in uni- and multicellular organisms such as bacteria, algae, protists, sponges, plants as well as almost all mammalian cells [2–6]. Via auto- and paracrine modes of action non-neuronal ACh promotes cell proliferation and differentiation as well as regulation of cell-cell contact, locomotion, transport of ions and water. Moreover, the reproductive system of mammals (sperm, granulosa cells, placenta, amniotic fluid) expresses components of the non-neuronal cholinergic system [3–6]. Non-neuronal ACh represents an extremely well conserved signaling molecule on the evolutionary time scale [3, 4]). While little is known about insects, ACh has been demonstrated in royal jelly, the secretion product of nursing bees for breeding [7]. This is consistent with the proliferative and so-called trophic effects of ACh mediated via muscarinic or nicotinic receptors, as has been repeatedly demonstrated elsewhere [2, 3, 6, 8–10].

The neuroactive neonicotinoids interfere with the cholinergic system by binding to the insect nicotinic receptors [11]. Although a causal relationship between bee losses and the qualified use of neonicotinoids in agriculture is still under debate, several studies discuss them as possible contributing factors for colony losses [12–17]. The increasing concern about their risks on bee health has led to an EU-wide ban by the European Commission on three commonly used neonicotinoids (clothianidin, imidacloprid, thiamethoxam). In this report we describe a so far unknown role for ACh in the breeding of honeybees and adverse effects of clothianidin and thiacloprid on this system.

## Materials and Methods

### Measurement of acetylcholine (ACh), choline-acetyltransferase (ChAT) activity and smooth muscle contraction

Acetylcholine was measured by cationic exchange high-pressure liquid chromatography (HPLC), combined with bioreactors and electrochemical detection [18]. ChAT enzyme activity was measured as described previously [19]. The longitudinal smooth muscle of the guinea pig small intestine was used and contractile responses were recorded as described elsewhere [20]. Guinea pigs were anesthetized with pentobarbital and sacrificed with carbon dioxide in compliance with the German protection of animals act.

### Western blot analysis (ChAT)

Extracted proteins of the hypopharyngeal gland were loaded on 10% polyacrylamide gels (15–20μg protein/lane), separated by SDS-PAGE in a vertical electrophoresis system (Mini-PROTEAN^®^ Tetra Cell) and subsequently transferred to a nitrocellulose membrane by Trans-Blot Turbo Blotting System^®^ (Biorad, Germany). After exposure to blocking buffer and overnight incubation at 4°C with the primary antibody (rabbit polyclonal anti-choline acetyltransferase, Abcam, ab68779, 10μg/ml), a corresponding HRP-conjugated secondary antibody (goat anti-rabbit; Jackson-Immunoresearch, Code 111-035-003) was applied. Proteins were visualized by Super Signal^®^ West Dura Extended Detection Kit (Thermo Scientific).

### Immunohistochemistry, scanning electron microscopy and immunogold electron microscopy

For immunohistochemical analyses specimens were placed in ice-cold isopentane, stored at -80°C and followed by standardized procedures to produce paraffin blocks and paraffin sections of 2 μm thickness. Immunohistochemistry was performed according to standardized methods in a fully automated manner (Dako Autostainer plus, Dako, Germany) by use of a 1:400 dilution for the first rabbit anti-ChAT antibody (Abcam ab68779, UK; second ab: Envision Flex+rabbit, Dako K8019, Germany). Histology was evaluated and documented using a Keyence BZ-9000E Hs all-in-one microscope (Keyence, Germany). For immunogold transmission electron microscopy specimens were fixed in buffered formaldehyde (3.7%) for 2 h at room temperature and stored overnight in phosphate-buffered saline at 4°C. Fixed specimens were embedded in epoxide, followed by the use of a standard protocol with application of the first antibody (ab68779) in a dilution of 1:100 and the second gold-labeled anti-rabbit antibody (Sigma Aldrich, G7402) in a dilution of 1:20. For scanning electron microscopy specimens were fixed in 2.5vol% glutaraldehyde (6 hours at room temperature). The analyses and photo-documentation were performed by using a transmission electron microscope (JEM-1400, Jeol, Tokio, Japan). Negative control experiments without the primary antibody were always carried out in parallel.

### Gene expression of ChAT and nicotine receptor subunits in the hypopharyngeal gland

Hypopharyngeal glands (3–4 pairs) of nursing bees were isolated, lysed and digested overnight at 56°C using Proteinase K (Roche Applied Science, Mannheim, Germany). Subsequently, alcoholic nucleic acid precipitation was performed and RNA purified by the commercial GenEluteTM Mammalian Total RNA Miniprep Kit (Sigma-Aldrich, Munic, Germany). Extracted RNA (1μg) was transcribed into cDNA according to a standard protocol using Omniscript RT PCR Kit (Quiagen, Hilden, Germany). The cDNA was amplified using gene-specific primers using Taq PCR Core Kit (Quiagen, Hilden, Germany). RT-PCR analysis was carried out using the following cycler program: 95°C for 10 min, 95°C for 30 s, 60°C for 30s, 72°C for 30 s, 72°C for 10 min, 40 cycles (GENEAMP PCR System, Applied Biosystems, Applera Deutschland GmbH, Darmstadt, Germany). 20 ng of cDNA were applied for each reaction together with 0.8 pmol/μl of one primer. The following gene-specific primers were used: reference gene Krh1 according to reference [21] (for: 5’-ACT CAT CAG TTG TTG GTT CTC CTC-3’; rev: 5’-TCG TTT GGC TCT TCA GTC TTG TG-3’), subunit of the insect nicotinic receptor α1 (for: 5’-TTC TCT CGG TGC TGG TGT TC-3’; rev: 5’-TTC ACG TTC AGC ACG GCT AT-3’; gene ID 726214, according to NCBI reference), α2 (for: 5’-ACA ATC GAC TGA TAC GCC CC-3’; rev: 5’-TGA TCC TGC CAT TCG TGC TC-3’; ID 406153), α3 (for: 5’-CAT CGC TCG TAG TAC CGC TT-3’; rev: 5’-TAT CTG GTA TTG CGG CCT GG-3’; ID 725996), α4 (for: 5’CGC AGA ACA CAC GAA AAT GC-3’; rev: 5’-TTG TGG CGG ACA GTT TAC CA-3’; ID 724143), α7 (for: 5’-CGC TGC GAG ATG AAA TTC GG-3’; rev: 5’- TTT TAC CAG GCACC CCC AAC-3’; ID 406148), α9 (for: 5’-CGT CCC ATC AAA TCG CCA AC-3’; rev: 5’- CGT CAT GTC GCC TGA ATT GT-3’; ID 411303) and choline acetyltransferase ChAT (for: 5’-TGA AAA GAC CAG CTT GGG CA-3’; rev: 5’-GAA AAT CGC TGG ACC TTC GC-3’; ID 100868164). The PCR products were separated by gel electrophoresis (2% agarose) and detected by ethidium bromide staining. Water controls and RNA sample controls were performed in parallel and were always negative. Primers were synthesized and purchased from Microsynth (Balgach, Switzerland).

### Removal of ACh from the larval diet and artificial larval breeding

Larval diet consisted of 50% (w/w) royal jelly (Imkerei Ullmann, Erlensee, Germany), 6% (w/v) glucose (Sigma Aldrich, Germany), 6% (w/v) fructose (Carl Roth GmbH, Karlsruhe, Germany) in sterile deionized water. Multiple protocols were tested to remove acetylcholine from this larval diet: different ionic strength and quantity of alkali to increase pH; application of acid at the end of the procedure to restore the original pH of 4; bolus or repetitive application of the alkali; use of acetylcholinesterase or butyrylcholinesterase, bolus or repetitive application of esterase; storage of the larval diet at room temperature or at 35°C. The following protocol proved to be effective: storage at 4°C during the procedure of alkaline treatment; application of 0.18–0.20% (vol/vol) 4 M NaOH at day 1 and 0.06–0.08% at day 2, obtaining a pH value of 5.5 (stronger alkaline treatment of the diet caused rapid death of the larvae). Thereafter, the modified diet was stored in 2 ml vials and 2.0 U/ml butyrylcholinesterase (acetylcholinesterase proved to be ineffective at a pH of 5.5) were added 2 or 3 times at 2 to 9 days intervals depending on the elimination rate of ACh. The 2 ml samples were stored at 35°C for a total period of 12 to 28 days according to the spontaneous fading of esterase activity at this temperature. The activity level of butyrylcholinesterase was tested at the end of the incubation period by adding external ACh to an aliquot of the stored samples. The probes were used as modified larval diet, when the ACh content did not decline within the next 24 h. Thus, a major butyrylcholinesterase activity was excluded, but some minor residual enzyme activity may still exist, when this modified diet was applied later on to the larvae. Applying this protocol allows a 75%-90% elimination of ACh. At the end of the incubation period half of the samples were supplemented with ACh to obtain a concentration of 6 mM, and the remaining samples received a corresponding water volume (6 μl). As ACh was applied in a minute volume (6 μl) of water the manipulated pH of the modified diet was not affected. Non-modified diet was used as control. Honeybee larvae were reared in 24-well tissue plates according to reference [22]. First instar larvae (ca. 12h, larval age was estimated by size) grafted from worker brood cells were used throughout the experiments. The larvae were carefully placed into a 24-well tissue culture plate (3.4 ml/well Orange Scientific, B-1420 Braine-l'Alleud, Belgium), each well was pre-filled with 100μl of the larval diet and thereafter incubated at 34.0°C and 97% humidity. Larvae were transferred every 48 h to a fresh well with new diet. To test the effect of ACh on survival larvae were fed with the ACh-supplemented and the non-supplemented modified diet. Control experiments using untreated diet were run in parallel.

### Long term exposure experiments to clothianidin or thiacloprid

Experiments were performed under semi-field conditions (September-October 2014; April-September 2015). No specific permission for the semi-field experiments with small bee colonies was required, because the area where the bee hives were placed belongs to the Institut für Bienenkunde Oberursel. No endangered or protected species were involved in this study. Small colonies (freshly used Mini Plus Beute) with 2,000–2,500 bees each, were placed outside in experimental tunnels. Each colony was loaded with apiary-mated sister queens (raised by material from the Institut für Bienenkunde) with certificate of descent. Each tunnel was equipped with two colonies and one treatment per tunnel was performed: clothianidin (1, 10 and 100 ppb), thiacloprid (200 and 8,800 ppb) or control. Neonicotinoids were pre-dissolved in acetone and finally dissolved in Apiinvert^®^ (sugar syrup consisting of 39 vol% fructose, 31 vol% saccharose and 30 vol% glucose; Südzucker AG, Mannheim, Germany) and fed via in-hive feeders. The final concentration of acetone in the sugar syrup was 100 ppm and this acetone concentration was also present in the control. Pollen (commercial biopollen of the company Seip, Butzbach, Germany) was offered *ad libitum* inside the tunnel for collection by forager bees and was refreshed each day. After at least three weeks of exposure to permit one full development cycle of a worker bee, brood combs with capped cells were stored overnight in an incubator (34°C). To obtain age-marked bees, all emerged bees from these combs were color-labeled and transferred back to their colonies for 6–9 days. Sampling of brood food was done by means of a finely prepared wooden toothpick always from brood cells containing larger larvae with visible food inside the cells and these cells were randomly distributed along the brood areas. Several parameters were obtained to estimate the vitality and brood success of the Mini-Plus hives in the tunnels: The amount of brood combs (with brood cells on both sides) was counted at 9 time points between days 4 and 39 (interval 2–5 days). To compare the sizes of brood nest areas, both sides of the two brood combs with the largest brood areas of each hive were photographed (except those fed with 100 ppb clothianidin, because brood development was seriously compromised) at day 13/16 and 29 after placement. From the photographs the number of all capped cells per comb side was counted manually. Pharyngeal glands were microdissected from marked bees, which were manually collected from brood combs. To facilitate the dissection, collected bees were immobilized on ice, decapitated and pharyngeal glands were isolated under a binocular microscope and then transferred directly to the respective medium for the measurement of ACh content (500 μl of a mixture of formic acid in acetone [15/85 vol%/vol%]), and ACh synthesis (50mM Na_2_HPO_4_, 300 mM NaCl, 10 mM EDTA, 0.1 mM physostigmine, 8 mM choline chloride, 1 mM acetyl-CoA and 0.5–1.0 vol% Triton X-100 or Triton X-100-free) or for microscopic investigation (see above). Likewise the brains of the bees were dissected under a binocular microscope for subsequent ACh content determination.

### Statistical analysis

Results were expressed as mean value ± sem of *n* experiments. Statistical analysis was performed using GraphPad Prism 6. Distribution of the values was tested by the Shapiro-Wilk normality test. In the case of non-parametric distribution or n < 7 significance of differences (set at p<0.05) was calculated by the Mann-Whitney test (GraphPad Software, San Diego, California, USA). Larval survival was analysed by the mean times of death, estimated with the Kaplan-Meyer estimate. The survival rates of control and treatment groups were compared using a log-rank test (Mantel-Cox test) using SPSS Statistics version 20.0 (IBM, Chicago IL).

### Drugs and special chemicals

Mini-PROTEAN^®^Tetra Cell-, Trans-Blot Turbo Blotting-Systems and all biochemicals used for Western blot analysis were purchased from Biorad, Munich, Germany, except for anti-ChAT ab from Abcam, Cambridge and goat anti-rabbit secondary ab from Jackson Immunoresearch, Newmarket, UK. Acetylcholinesterase and butyrylcholinesterase were purchased from Sigma-Aldrich, Munich, Germany. Pierce BCA Protein Assay Kit and Super Signal West Dura Extended Detection Kit were from Thermo Scientific, Waltham, US. All other drugs and biochemicals, including clothianidin and thiacloprid, were purchased from Sigma-Aldrich, Munich, Germany.

## Results

### ACh is synthesized within the hypopharyngeal gland by membrane-bound ChAT

**Fig 1:**
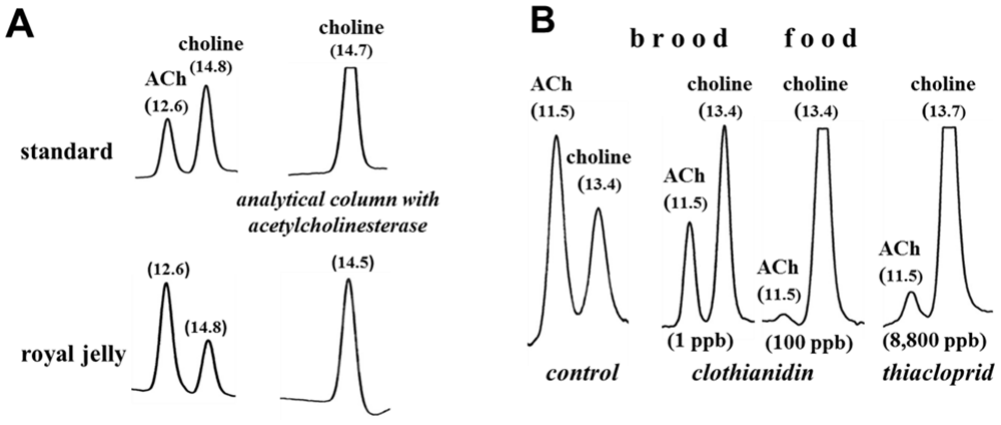
HPLC measurement of ACh/choline in royal jelly (A) and brood food (B). A: Typical chromatograms after injections of 1 pmol ACh and choline (standard) or of an aliquot (20 μl) of 12 mg royal jelly diluted in 1 ml HPLC buffer and further diluted by a factor of 1000. Chromatograms on the right hand show the same samples using an analytical column packed with acetylcholinesterase, i.e. ACh is already hydrolyzed during its passage along the analytical column; thus the ACh peak disappears and the choline peak increases. Retention times are indicated in brackets. B: Chromatograms of brood food collected from semi-field control (4.5 mg, diluted by 500) or from a hive exposed to 1ppb (1.6 mg brood in 500μl, diluted by 200), 100 ppb clothianidin (1.4 mg; diluted by 100) or to 8,800 thiacloprid (1.3 mg; diluted by 100); 2 x zoom out compared to 1A.

It has already been reported that ACh is present in royal jelly [RJ] (683–800 μg/g), the secretion product of nursing bees to feed their queen and worker brood [7]. This value correlates with the present values of ACh measured by means of HPLC, combined with enzymatic bioreactors and electrochemical detection (Fig 1A, Table 1A). We found up to 8 mM ACh (corresponding to about 1400 μg/g) in RJ freshly isolated from queen cells just 2–3 h after the nursing of fertilized eggs. A comparable amount was detected in RJ collected from queen cells occupied by 3 to 7 day old queen larvae, in the worker brood food or in commercially available RJ purchased from a company in China (see Table 1A and Fig 1B [control]). In contrast, the honeybee brain contained only about 230±50 nmol/g ACh (Table 1A). Comparable to the brain, the amount of ACh in RJ exceeded that of choline (Fig 1A and 1B [control] and Table 1A), a very uncommon finding, because biological material outside the brain regularly contains more choline than ACh [23]. Commercial honey contained less, but still significant amounts of ACh (Table 1A).

**Table 1:** Content of acetylcholine (ACh) and choline and the ratio between both com-pounds in samples obtained from control bees (A; outside control) and from bees kept under semi-field condition to investigate the effect of neonicotinoids (B).

**A**	**ACh content**	**choline content**	**ratio ACh/choline**
**Food for queen larvae** (royal jelly)	4.64 ± 0.88 (10) mM	2.50 ± 0.18 mM	1.89 ± 0.36
**Freshly isolated royal jelly**	8.48 ± 2.93 (7) mM	2.36 ± 0.48 mM	4.45 ± 1.80
**Food** (working bee larvae, outside control)	4.13 ± 0.31 (31) mM	1.75 ± 0.20 mM	2.90 ± 0.33
**Hypopharyngeal gland**			
control	67 ± 37 nmol/g (11)	533 ± 115 nmol/g	0.11 ± 0.06
exposed to neonicotinoids)§	37 ± 16 nmol/g (5)	884 ± 295 nmol/g	0.04 ± 0.01
**Brain**	230 ± 50 nmol/g (10)	63 ± 20 nmol/g	6.60 ± 1.70
**Fertilized egg**	23 ± 20 pmol/egg (3)	800 ± 250 pmol/egg	0.02 ± 0.01
**Commercial honey**	24 ± 11 μM (6)	120 ± 22 μM	0.30 ± 0.10
**B**			
**Food** (working bee larvae)			
**Semi-field control**	2.67 ± 0.49 (24) mM*	2.05 ± 0.23 mM	1.68 ± 0.32*
**Clothianidin**: 1 ppb	1.72 ± 0.25 (17) mM	1.88 ± 0.18 mM	1.07 ± 0.17
10 ppb	1.88 ± 0.26 (16) mM	2.16 ± 0.41 mM	1.31 ± 0.37
100 ppb	0.57 ± 0.18 (6) mM^##^	1.76 ± 0.57 mM	0.55 ± 0.23^#^
**Thiacloprid**: 200 ppb	1.31 ± 0.28 (10) mM^#^	3.18 ± 0.49 mM^#^	0.45 ± 0.08^##^
8,800 ppb	0.81 ± 0.41 (3) mM	5.38 ±1.63 mM	0.21 ± 0.14

Royal jelly or brood food were diluted in HPLC buffer and tissue was homogenized in a mixture of formic acid and acetone (see Methods); between 0.5–12 mg of royal jelly/brood food, 2–3 pairs of the hypopharyngeal gland and 5–7 eggs were used for an individual sample. Aliquots of the diluted samples were injected into the HPLC and content of ACh and choline quantified by comparison with external standards of ACh and choline and standardized to 1g or 1 egg. Means ± sem of the number of experiments indicated in brackets.

^§^: Pooled data obtained with 8,800 ppb thiacloprid (n = 3) and 100 ppb clothianidin (n = 2). Significance of differences: between outside control and semi-field control

* (p<0.01); between semi-field control and neonicotinoid-exposed bees,

^#^ (p<0.05),

^##^ (p<0.01).

**Fig 2:**
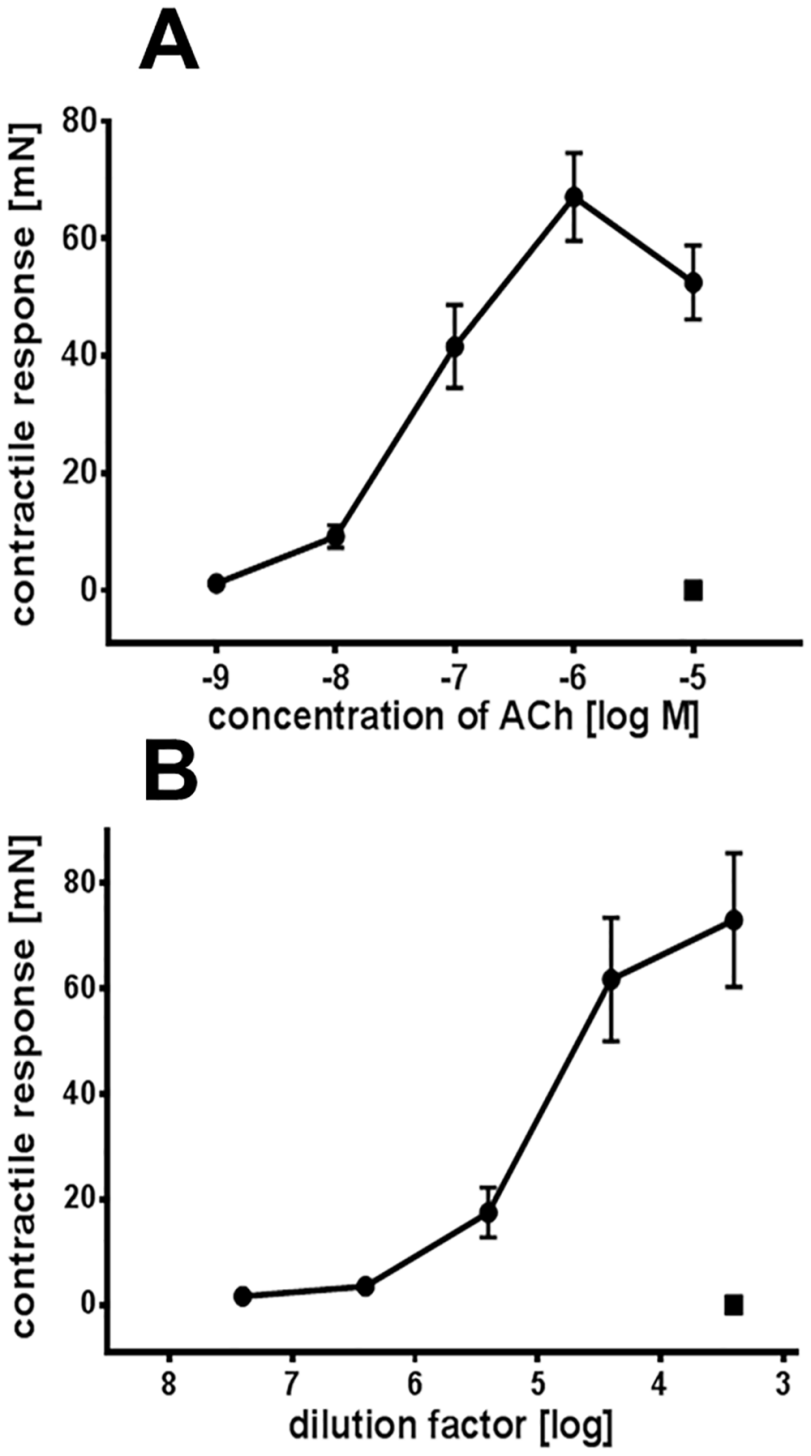
Longitudinal smooth muscle contraction of the guinea pig small intestine. A: The dose-response curve for ACh. B: The dose response curve for different dilutions of royal jelly. Each curve shows the means ± sem of 5 experiments; squares: presence of 1 μM atropine.The source of ACh synthesis responsible for its presence in brood food has not been identified so far [7]. We therefore investigated the ACh content and *in vitro* synthesis of ACh, i.e. the activity of the synthesizing enzyme choline acetyltransferase (ChAT) in homogenates of the hypopharyngeal glands of nursing bees by using a standard buffer containing the detergent Triton X-100. Only a low ChAT activity of 0.08±0.06 nmol/mg protein/h was found (Table 2). In subsequent experiments hypopharyngeal glands were placed in a non-homogenized state in an assay buffer free of Triton X-100. Under these conditions the enzyme activity increased nearly 30,000-fold to 2.2±0.80 μmol/mg/h and bromoacetylcholine (30 μM), an inhibitor of ChAT, reduced ChAT activity by 90% (Table 2).

The biological activity of ACh within RJ was investigated using the isolated longitudinal smooth muscle preparation of the guinea-pig ileum (Fig 2). RJ at a dilution as high as 6.0 log units induced a weak contraction. The maximum effect was observed at a 3.4 log unit dilution (representing a concentration of 1.3±0.14μM ACh) which corresponded to the effect of 1μM standard ACh (Fig 2). The contractile response of RJ was completely blocked by 1μM of the muscarinic receptor antagonist atropine (Fig 2), thus demonstrating the cholinergic biological activity of ACh present within RJ. This confirms that RJ can also produce spasmogenic effects.

**Table 2:** ChAT activity and ACh release obtained with hypopharyngeal glands isolated from outside control bees.

Hypopharyngeal gland	ChAT activity	ACh release
(outside control)	(nmol/mg protein/h)	(nmol ACh/30 min/90μl)
**Nursing bees**		506 ± 102 (5)
presence of Triton X-100 (1.0 vol%)	0.08 ± 0.06 (n = 8)	
absence of Triton X-100	2.2 ± 0.80 (n = 9)*	
presence of bromoacetylcholine (30 μM)	0.19 ± 0.07 (n = 3)^§^	
**Worker bees**		171 ± 33 (10)#

Hypopharyngeal glands were dissected and ChAT activity and ACh release determined as described in Methods. Upon omission of Triton X-100, i.e. membrane-bound proteins remained, ChAT activity increased roughly 30,000 fold

(* p<0.001). The ChAT inhibitor bromoacetylcholine reduced enzyme activity by 90%

(^§^ p<0.01). Hypopharyngeal glands isolated from nursing bees showed a 3fold higher ACh release than worker bees which were collected before the hive entrance

(^#^ p<0.01). Shown are means ± sem of the number of experiments indicated in brackets.

**Fig 3:**
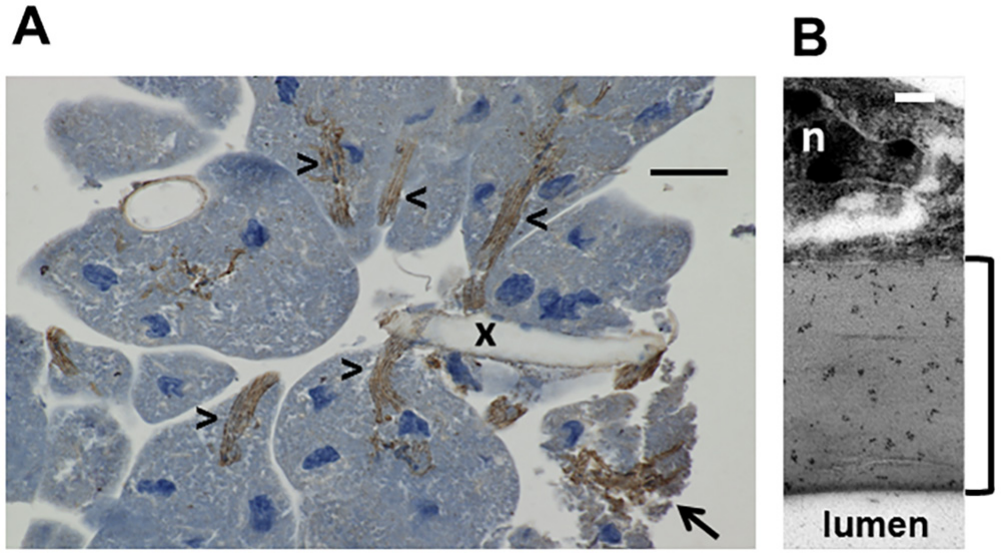
Histological analysis of the hypopharyngeal gland by anti-ChAT immuno-histochemistry and immunogold transmission electron microscopy. A: Control: secretory acini with cytosol and nuclei (blue staining) and a tangentially cut collecting duct (x) are shown. The fine long distance microtubes (arrowheads) of the so-called canal cells (nuclei not visible in the image), which open into the collecting duct (x), show prominent anti-ChAT immunoreactivity (brown staining). The scaffold of the canal cells is even maintained in acini damaged as a result of the preparation procedure (arrow); bar represents 40 μm; 5 glands were investigated in parallel. B: Transverse section of the wall of the large collecting duct investigated by immunogold transmission electron microscopy. The lumen is located at the bottom and the outer coating (adventitia) shows a portion of the nucleus (n) at the top of the image. Significant deposition of gold particles (dark points) is detected within the luminal part of the duct wall (see bracket); bar represents 200 nm.

**Fig 4:**
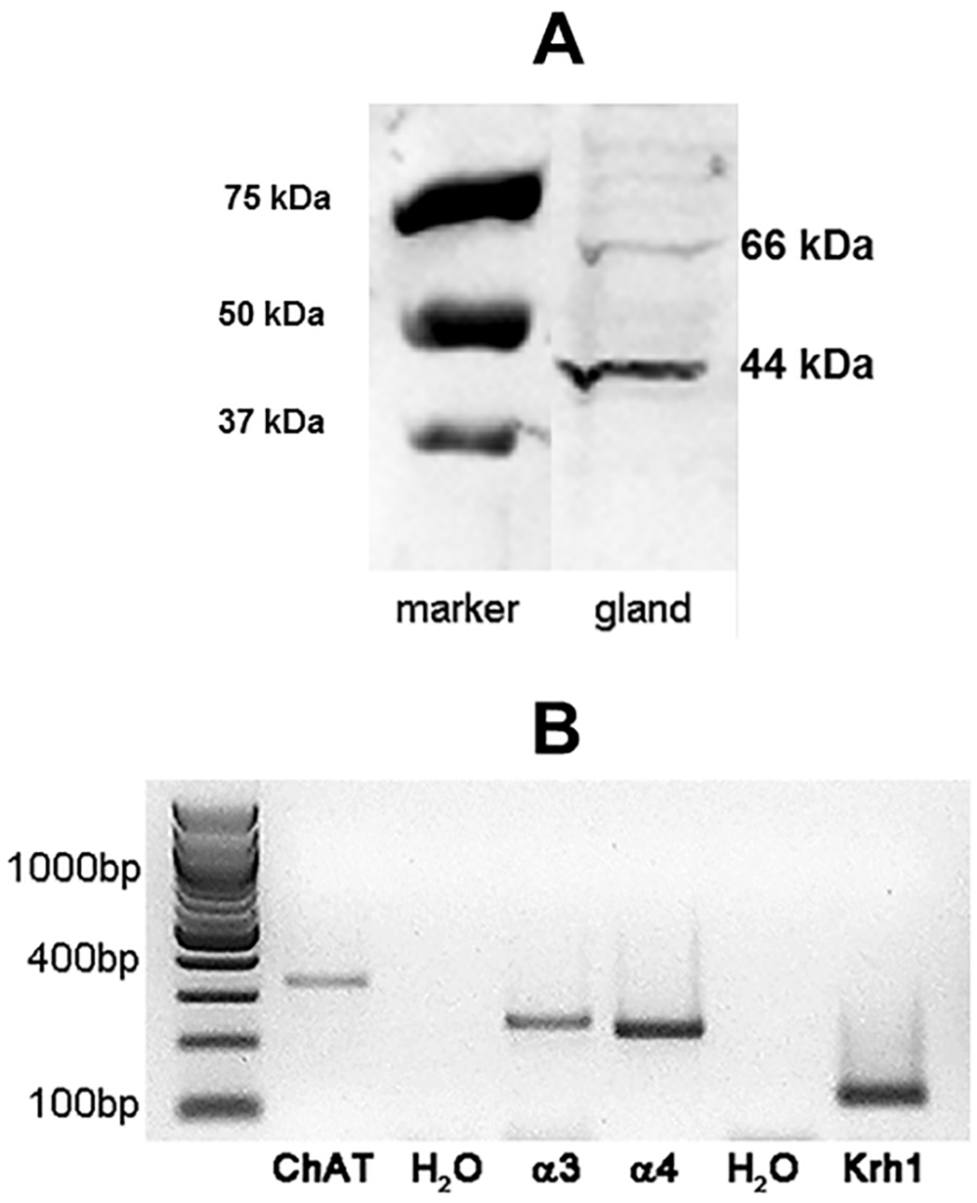
Demonstration of ChAT by Western blot and expression of ChAT gene and genes of the nicotinic receptor subunits within the hypopharyngeal gland. A: Western blot of proteins extracted from hypopharyngeal gland using anti-ChAT ab showing two bands at 44 and 66 kDa. B: Expression of the ChAT gene (expected size of the amplification product: 332bp) and α3- (239bp) and α4-nicotinic (226bp) receptor subunit gene in the hypopharyngeal gland demonstrated by RT-PCR using gene-specific primers; negative results were obtained with the primers specific for α1, α2, α7 and α9 (not shown). The reference gene Krh1 (118 bp) was always positive and the water control negative. Exemplary results of 4 different preparations are shown.

Despite the high ChAT activity, freshly isolated hypopharyngeal glands contained only 67±37 nmol ACh/g wet weight (Table 1A), indicating that ACh is not stored in the glands but immediately released into RJ/brood food following its synthesis. This pathway is strongly supported by the localization of the ChAT enzyme within the gland as demonstrated by anti-ChAT immunohistochemistry (Fig 3A). The secretory acini produce the brood food, which is transported via the canal cells to the collecting duct, i.e. these microtubes link the secretory cells to the collecting duct to transport their acidic secretions [24]. Using anti-ChAT antibody hot spots were visualized within the walls of these canal cells and within the inner wall of the collecting duct (Fig 3A and 3B). Thus, anti-ChAT immunoreactivity showed a membrane-bound pattern which corresponds with the biochemical measurement of ChAT enzyme activity. Expression of the ChAT gene was demonstrated by RT-PCR experiments and ChAT-protein was detected by Western blot (Fig 4A and 4B), showing bands at 44 and 66 kDa, which may correspond to the so-called p-ChAT isoform (molecular weight in mammals about 49 kDa) and to the well-studied 67 kDa c-ChAT.

### ACh is extremely well conserved in brood food by an acidic pH and is essential for larval breeding

**Fig 5:**
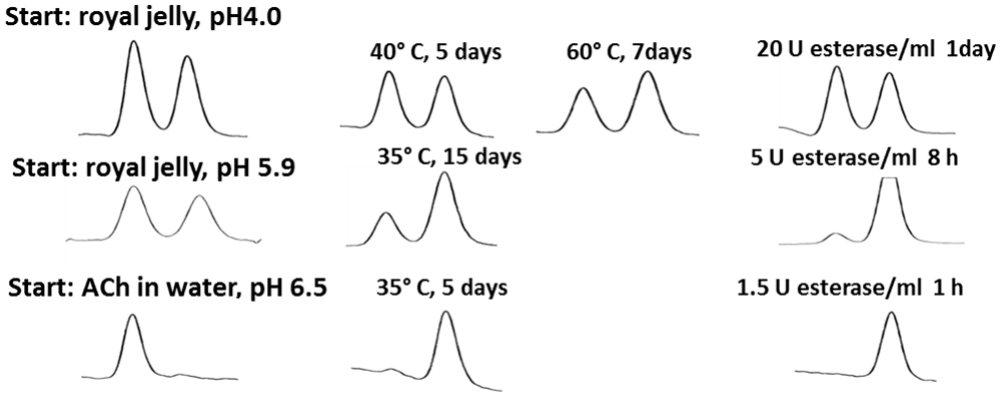
HPLC measurement of ACh and choline in royal jelly and water after incubation at different pH values and application of esterase. Aliquots of royal jelly were stored at the conditions indicated and analysed by HPLC. For comparison, a water solution containing 5 mM ACh was also investigated. The first peak corresponds to ACh and the second to choline. Reduction of the first peak and increase of the second one reflects hydrolysis of ACh. Typical chromatograms are presented (n = 3).

RJ is characterized by an acidic pH of about 4, a condition that protects ACh from degradation. Neither heating (40–60°C) nor the application of the degrading enzyme acetylcholinesterase reduced the ACh content in RJ (Fig 5). By contrast, ACh was more or less completely eliminated in an aqueous solution with a pH above 6 (Fig 5). When the pH of RJ was shifted to 5.9 by titration with small amounts of 4 M NaOH (in total 0.28–0.30 vol%), warming to 35°C or applying acetylcholinesterase resulted in reduced ACh contents (Fig 5).

**Fig 6:**
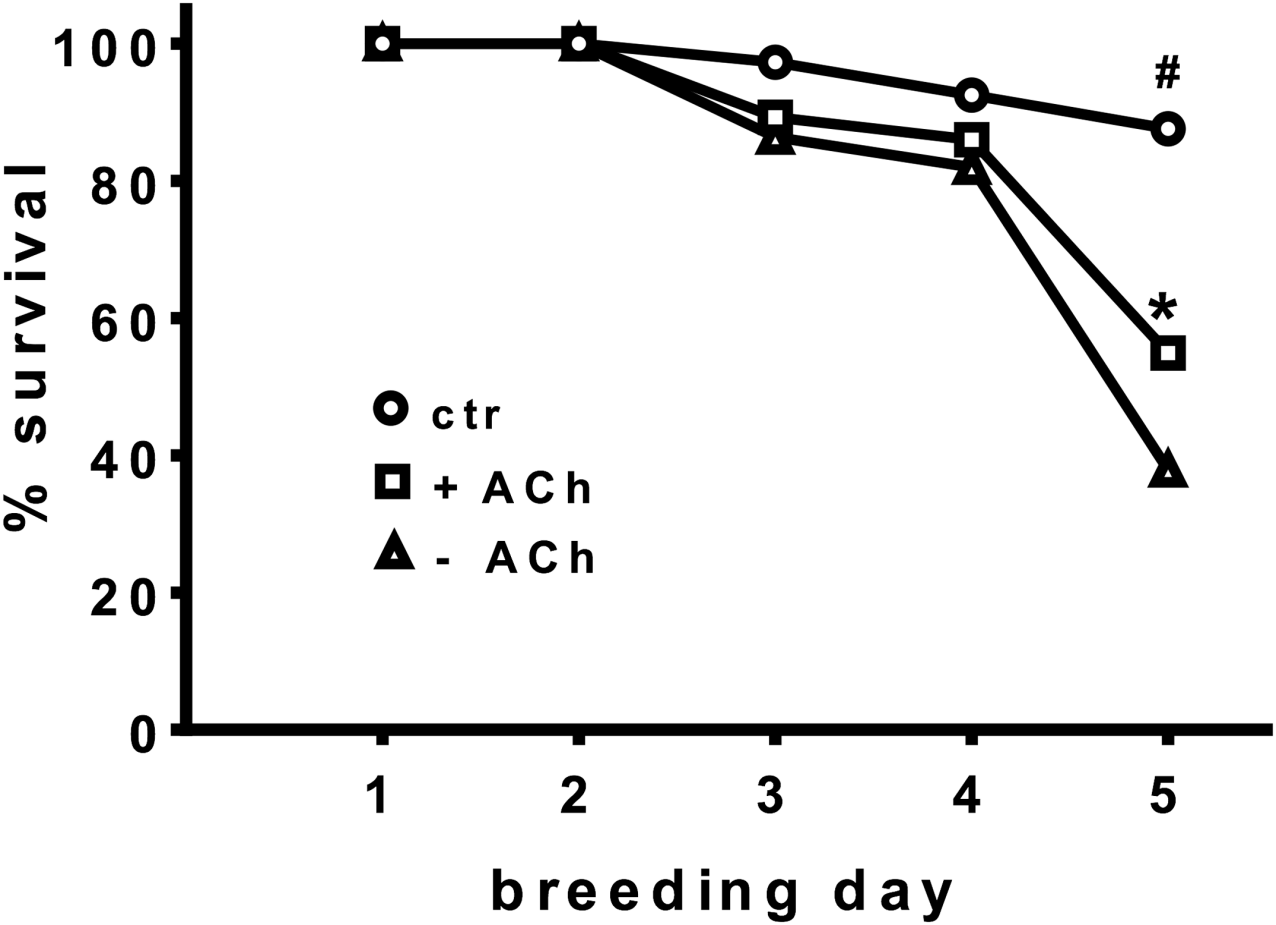
Effect of modified larval diet with reduced or supplemented ACh content on artificial larval breeding. Larval diet was pre-treated by the application of NaOH (to increase the pH from 4 to 5.5) and of butyrylcholinesterase to remove ACh (see Methods), followed by storage at 35°C for 12–28 days, which led to the removal of about 75–90% of ACh (-ACh). For comparison, aliquots of this modified diet were supplemented with 6 mM ACh (+ACh) before starting artificial larval breeding and non-modified diet was used as control (ctr). Larvae were reared in 24-well plates with fresh diet every 2 days. Larval survival rate during the first 5 days is shown. After this period more or less all larvae fed with the modified diet were dead, most likely because of the compromising pre-treatment. The results of 4 different series of experiments were pooled (control: 186 individual larvae in 4 series of experiments; +ACh: 206; -ACh: 177) and compared by mean time of death, estimated with the Kaplan-Meyer estimate and compared with log-rank test, Mantel-Cox test. In each of the 4 series +ACh larvae showed a higher survival rate than –ACh larvae (12.8%; 35%; 15.2%; 6.3%); * difference between +ACh and –ACh, p<0.01; # difference between both treated groups and the control, p<0.01.

Feeding such a modified RJ (pH 5.9) to artificially bred larvae caused larval death within 2–4 days (data not shown). Applying a more discreet protocol to remove ACh from the larval food (application of 4 M NaOH in 2 steps and increasing the pH maximally to 5.5; stepwise application of butyrylcholinesterase; see Methods) offered the possibility to test the effect of low ACh content vs. ACh supplementation in artificially bred larvae; non-modified larval food was used as control (Fig 6). As a consequence of the critical manipulations (adding 4 M NaOH and butyrylcholinesterase) the brood survival rate was substantially lowered compared to the control. Nevertheless, despite this fact, ACh-supplementation increased the survival rate significantly at day 5 (Fig 6), thus demonstrating the relevance of ACh as one essential component for breeding. The importance of ACh for breeding is further indicated by the observation that the capacity of the hypopharyngeal gland to produce ACh is about 3 times higher in nursing than in worker bees (506±102 nmol ACh/30 min/90μl vs 171±33; Table 2).

### Chronic exposure of *Apis mellifera carnica* colonies to neonicotinoids compromises ACh-synthesis

**Fig 7:**
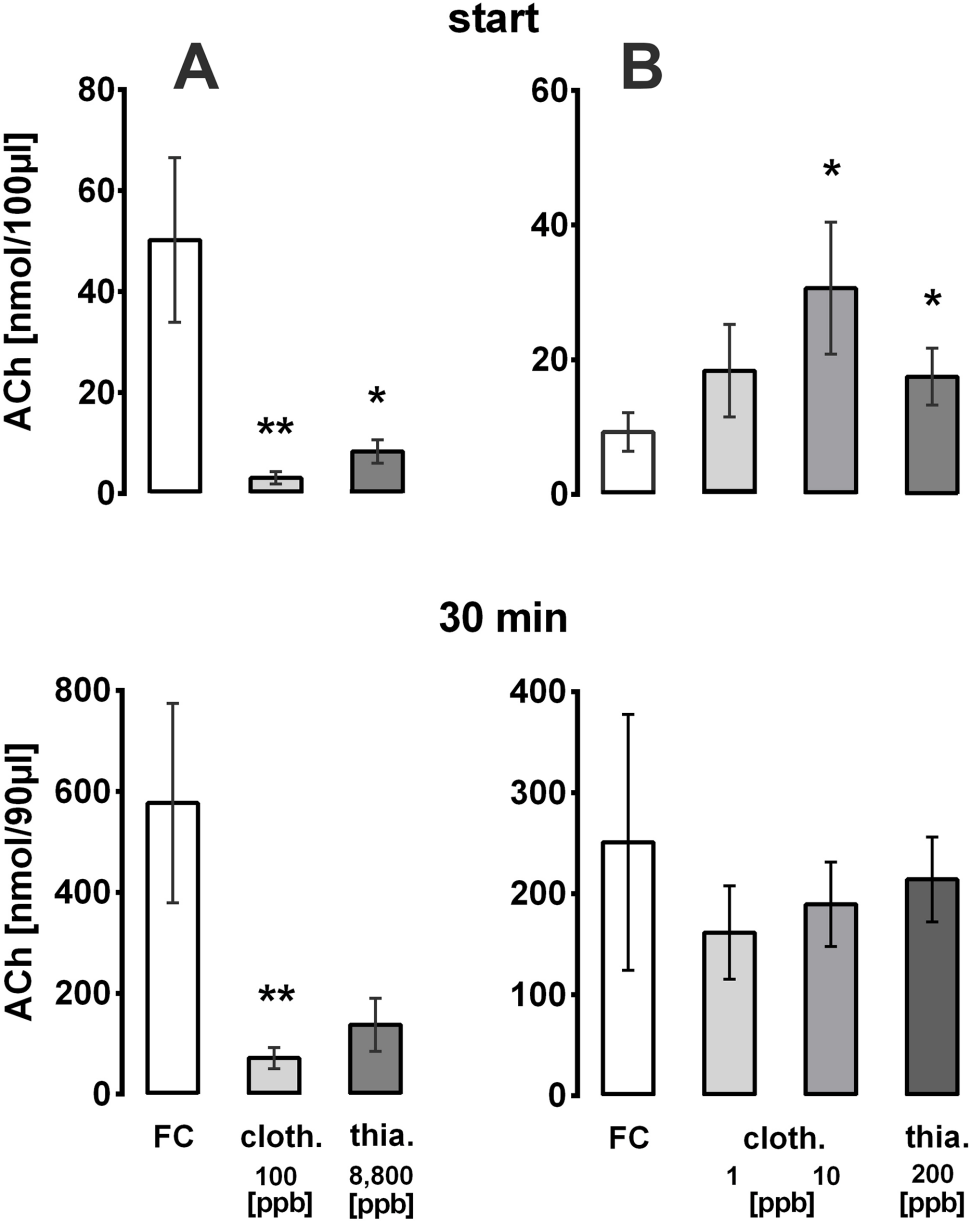
HPLC measurement of ACh release from hypopharyngeal glands under control conditions (FC) or after long term exposure of honeybees to neonicotinoids. The release of ACh from hypopharyngeal glands (3 pairs per individual sample) dissected from semi-field control bees (FC) or neonicotinoid-exposed bees is shown (cloth: clothianidin; thia: thiacloprid). An aliquot (10 μl) was removed from the assay buffer at the start and end of the 30 min period. Given are means±sem of the ACh content at both time periods A: Experiments were performed during September-October 2014; n = 9 for control and n = 6 for each neonicotinoid. B: Experiments were performed during April-September 2015; n = 15 for control and n = 7–11 for each neonicotinoid.). Significance of difference from the FC: * p<0.05;** p<0.005.

Hives were chronically exposed over a 4 week period to either clothianidin (1, 10 or 100 ppb in sugar sirup; 4 hives for each dose) or thiacloprid (200 or 8,800 ppb; 2 hives for each dose). After the insecticide exposure brood food was sampled from brood cells of these hives and the ACh amount measured by HPLC. Clothianidin (100 ppb) and thiacloprid (8,800 ppb) reduced the ACh content maximally by about 80%, whereas the choline content increased (Table 1B and Fig 1B). It should also be noticed that the ACh content and ratio ACh/choline declined significantly in the semi-field control compared to the outside control hives, thus indicating a compromising situation for the bees placed for several weeks in flight tunnels (Table 1A and 1B). An additional explanation could be the presence of 100 ppm acetone in the sugar syrup. Despite this condition, low doses of clothianidin (1 and 10 ppb) or 200 ppb thiacloprid showed an additional reduction of the ACh content and of the ACh/choline ratio, which in the case of 200 ppb thiacloprid was statistically significant (Table 1B). The ACh content of hypopharyngeal glands isolated from neonicotinoid-exposed bees (high doses) did not differ significantly from unexposed controls, however 50% less ACh and 60% more choline were found (Table 1A). This finding excludes an enhanced glandular storage of ACh to be responsible for the reduced levels of ACh in the brood food. Consequently, the synthesis and release of ACh may be impaired by neonicotinoids. To test this, hypopharyngeal glands of 6–9 day old nursing bees of the semi-field control as well as of neonicotinoid-treated hives (100 ppb clothianidin; 8,800 ppb thiacloprid; experiments performed during September-October 2014) were dissected and placed in an incubation medium to measure the release of ACh during a 30 min period. Corresponding with the decline in the brood food we found an 85% reduction of ACh release from the hypopharyngeal glands isolated from bees exposed to the high doses (Fig 7A). The effects of lower doses (1 and 10 ppb clothianidin; 200 ppb thiacloprid) were investigated during April-September 2015 (Fig 7B). After a 30 min exposure to the assay buffer the release of ACh from the glands dissected from bees exposed to the lower neonicotinoid doses (1 and 10 ppb clothianidin; 200 ppb thiacloprid) was always lower (161.5±46.2 nmol/90 μl; 189.4±41.7; 214.0±42.3) than that of the control glands (250.8±126.7), but the differences were not statistically significant (Fig 7B). In these experiments the start release of neonicotinoid-exposed glands was higher than that of the respective field control (Fig 7B), which may reflect a compensatory mechanism, to attain at least the somewhat smaller levels of ACh detected in the present experiments.

**Fig 8:**
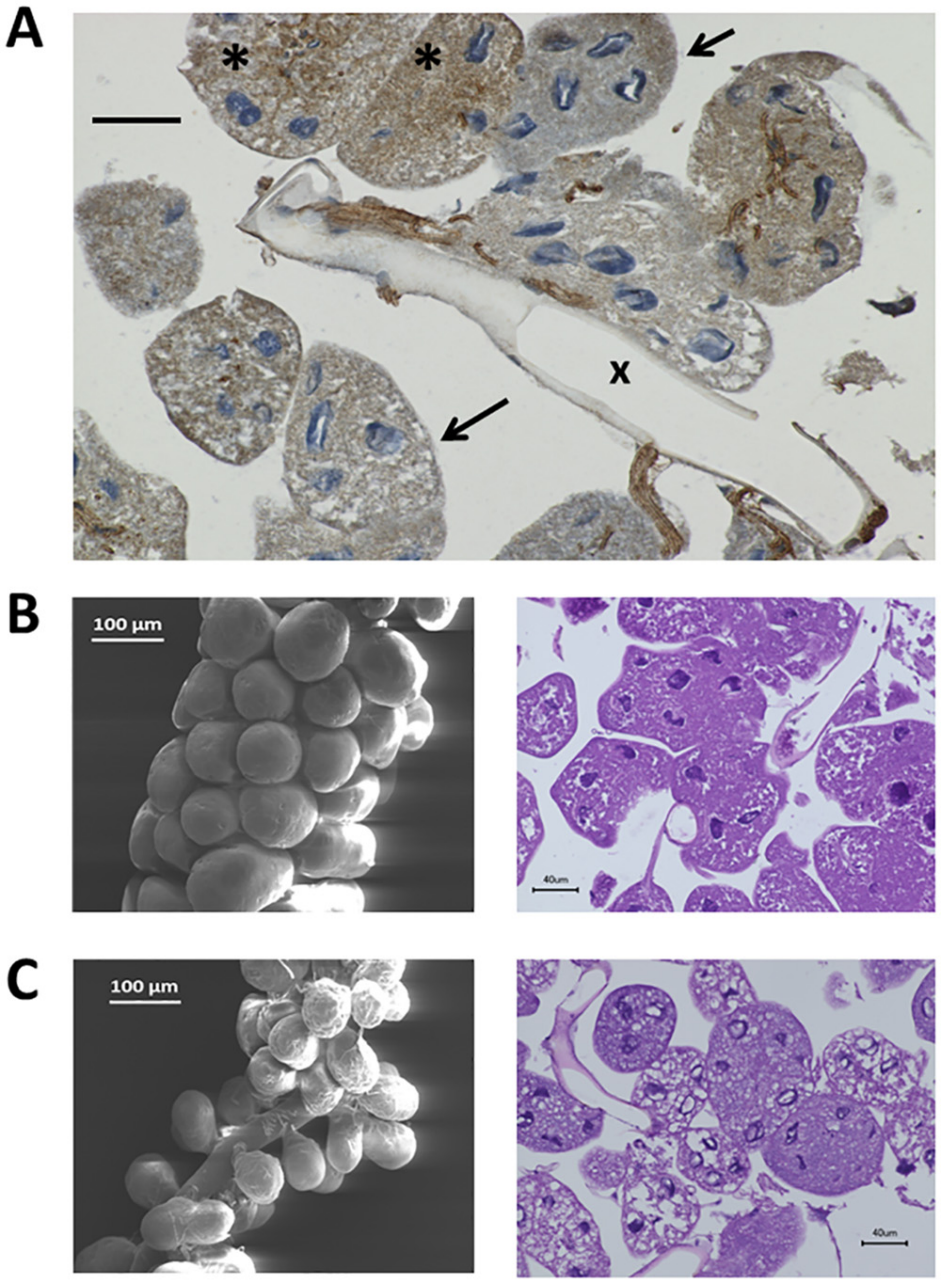
Histological analysis of the hypopharyngeal gland by anti-ChAT immunohistochemistry, scanning electron microscopy and haematoxylin-eosin staining under control (B) or after long-term exposure to neonicotinoids (A,C). A: Exemplary image of the hypopharyngeal gland (4 glands were investigated) obtained from a nursing bee of a clothianidin-exposed hive (100 ppb); please compare with the image of the unexposed control in Fig 3A. There is reduced size of the acini, which are in different stages of regression, especially the canal cells. This results in their fragmentation with loss of their directionality toward the collecting duct (x) and granular distribution of the ChAT immunopositivity within the cytosol (*), which is markedly vacuolated compared with the control; some acini (arrows) do not contain any visible canal cells at all, whereas intact immunopositive canal cells are present near the collecting duct and demonstrate a disturbed orientation within cells (#). Bar represents 40μm. B: Control glands; scanning electron microscopy shows the secretory buds. These are arranged like dense, grape-like clusters along the main collecting duct, which is masked by the buds in B but becomes clearly visible in C; HE-staining demonstrates the large amount of cytosol of the secretory acini with their nuclei and a tangentially cut collecting duct. C: Glands isolated from bees exposed to 8,800 ppb thiacloprid (scanning) or to 100 ppb clothianidin (HE-staining). The ultrastructural images confirm the loss of the secretory buds in the latter case, while conventional histology illustrates marked cytoplasmic vacuolization of the acini.

The cellular mechanisms behind the reduction of the ACh synthesis remain to be investigated. We can exclude an acute effect of the neonicotinoids on nicotinic receptors expressed in the hypopharyngeal gland. Fig 4B shows the expression of the α3- and α4-subunit of the insect nicotinic receptor. These subunits can be expressed by the glandular cells or by crossing/ innervating neurons. A high dose of thiacloprid (2,500 ppb corresponding to 10 μM) applied directly to the assay buffer did not affect ACh release in comparison to the untreated outside control performed in parallel (587±240 nmol/90 min [n = 5] vs 506±102 [n = 5]). Therefore, a direct stimulation of nicotinic receptors does not affect the synthesis of ACh, but a chronic exposure of the bees and their hypopharyngeal glands to neonicotinoids is required to cause impaired ACh synthesis. Chronic exposure to high neonicotinoid doses caused vacuolisation and subsequent degradation of the secretory cells (compare Fig 8B and 8C). The ChAT-immunopositive canal cells showed regression and fragmentation, which would explain the reduced ACh secretion (compare Figs 3A and 8A). Secretory buds which were still connected to the collecting duct displayed a disturbed outer surface, thus underlining the cellular damage (Fig 8C). The loss of secretory cells is also substantiated by a reduced wet weight of neonicotinoid-exposed hypopharyngeal glands (100 ppb clothianidin: n = 2; 8,800 ppb thiacloprid: n = 3) as compared to glands from control bees (1.51±0.55 [n = 5] vs 5.69±0.89 mg/pair [n = 11]; p< 0.01).

### Chronic exposure of *Apis mellifera carnica* colonies to neonicotinoids compromises the larval development

Experiments were performed under semi-field conditions with small colonies (2,000–2,500 bees each) placed outside in experimental tunnels. Each tunnel was equipped with two colonies which received the same treatment per tunnel: control, clothianidin (1, 10 and 100 ppb) or thiacloprid (200 ppb). Brood development mirrored the reduced ACh levels in three ways:

1. In outside control hives the number of brood combs increased continuously and brood areas darkened constantly over 4 weeks and extended over almost the complete comb, which was not observed in combs from tunnel-placed hives. At day 13/16 an average of 250 capped cells (minimal value: 102, maximal value: 510; 8 frame sides from 4 frames of 4 colonies) was counted. The brood area of tunnel semi-field controls was smaller than the outside controls with an average of 83 (32,140; 6 frame sides from 4 frames of 4 colonies) capped cells on day 13/16. This correlates with the reduced ACh-levels and is in line with the stressor of the semi-field condition, i.e. placed for 4 weeks in tunnels.
2. Hives treated with 1 or 10 ppb clothianidin (6–8 frame sides from 4 frames of 4 colonies, i.e. 2 separate experiments using 2 colonies each) showed an average of 62 (0, 114) and 60 (15, 134) capped cells at day 13/16, respectively. Exclusively in the hives exposed to 1 ppb clothanidin repeated removal of eggs was observed. Hives treated with 200 ppb thiacloprid (n = 4 frame sides of n = 2 colonies) had an average of 70 (53, 87) capped cells at day 16. Compared to the semi-field control, the reduced number of capped cells correlates with the reduced ACh content in the brood food observed after chronic exposure to the low neonicotinoid doses (significant 50% reduction in the case of 200 ppb thiacloprid and non-significant 32% reduction in the case of clothianidin; see Table 1B). The delay of brood development was reversible, because after 4 weeks the neonicotinoid-exposed hives did not differ from the semi-field control.
3. Brood development of hives exposed to 100 ppb clothianidin (n = 2 hives) was severely compromised during the whole observation period. No capped brood cells were found until day 26 and brood cells mainly contained eggs that did not further develop into larvae. Taking all data together the three levels of compromised brood development observed corresponded with the reduced ACh content in the brood food.

## Discussion and Conclusion

The present experiments demonstrate the following findings:

1. The hypopharyngeal gland of nursing honeybees produces unusually high amounts of non-neuronal ACh, which results to millimolar ACh concentrations present in royal jelly and brood food.
2. ACh synthesis is mediated by membrane-bound ChAT, which is localized within the wall of the canal cells and the collecting tube. These canal cells represent cuticular microtubes which accumulate and transfer the secretion product to the collecting duct, which itself ends in the nursing bee’s mouthparts. The cuticular structure [24] is required because of the acidic secretion.
3. The acidic property of royal jelly/brood food protects non-neuronal ACh from spontaneous or enzymatic hydrolysis, thus ensuring 100% delivery of intact ACh to larvae.
4. Supplementation of manipulated larval food to remove endogenous ACh with 6 mM ACh significantly increases the survival rate compared to ACh-reduced larval food.
5. Within the hypopharyngeal gland α3 and α4 nicotine receptor subunits are expressed, which represent possible targets for neonicotinoids. A high dose of the neonicotinoid thiacloprid (10 μM; corresponding to 2,500 ppb) did not produce an acute effect on the synthesis of non-neuronal ACh, but impairment was observed following long-term exposure.
6. Exposure of small bee colonies under semi-field conditions to low doses of clothianidin (1 and 10 ppb) or thiacloprid (200 ppb) reduced the ACh content in larval food on average by 40%. High doses (100 ppb clothianidin, 8,800 ppb thiaclorid) reduced the ACh release from the hypopharyngeal gland and the ACh content in the brood food by 80%, i.e. a dose-response relationship was found. The high doses of both neonicotinoids damaged the synthesizing system in the gland. Moreover, the reduced ACh levels in the larval food correlated with a compromising effect on brood development.

The biological role of non-neuronal ACh has been delineated in the last 2 decades, showing its universal expression independent of neurons as well as its involvement in various cell functions [2–6, 25–27]. Particularly, so called trophic effects of ACh mediated via muscarinic or nicotinic receptors have been repeatedly demonstrated in the plant and animal kingdom [2–6, 8–10, 25–28]. These trophic effects may also be involved in the larval development of honeybees. The nursing bees have optimized the system by releasing ACh into an acidic micro-environment. This ensures that intact, non-hydrolised ACh, i.e. a percentage of 100%, is delivered directly to the larvae. Thus, the structural and functional organization already points to a relevant role of non-neuronal ACh for the breeding of honeybees.

The significance of ACh for larval breeding is also demonstrated by the present artificial breeding experiments. In order to test the role of ACh for larval development, ACh was removed from brood food. Artificial removal of ACh was possible only by adding 4 M NaOH to increase the pH from 4 to 5.5 and by exposure to unspecific esterase, i.e. by accepting severe manipulations with changes of the physico-chemical properties of the larval food. Butyrylcholinesterase, which can inactivate multiple bioamines, requires at least a pH of 5.5, as at lower pH values the enzyme is inactive. Nevertheless, despite these severe manipulations ACh-supplementation significantly increased the survival rate at day 5, under these hostile circumstances all the more remarkably. The significance of ACh for breeding is also supported by the present observation that the capacity of the hypopharyngeal gland for ACh synthesis was significantly higher in nurse than in worker bees. However, it is not known why such high concentrations are required, as typically nicotinic and muscarinic receptors will be stimulated by lower concentrations. Hence, one may speculate that millimolar concentrations of ACh also mediate effects independent of the nicotinic or muscarinic receptors. Thus, for example ACh represents an ammonium compound and this class is known to mediate anti-microbiological effects. In this context is should also be considered that the acidic pH of the brood food itself represents a conservation method to prevent bacterial contamination.

The present experiments were also designed to investigate the effects of neonicotinoids on non-neuronal ACh. This class of registered insecticides interacting with the cholinergic system, i.e. the insect nicotinic receptors [11], is frequently used world-wide in agriculture and has been successfully applied to control pests in agricultural crops. However, the use of these compounds has been increasingly criticized on account of their potential adverse environmental effects. In particular, it has been criticized that the neonicotinoids compromise non-target organisms, such as insect pollinators and may at least contribute to honeybee losses [12–17, 29–34]. The present experiments demonstrate the expression of nicotine receptors subunits as possible targets for these insecticides to mediate adverse effects on non-neuronal ACh synthesized in the hypopharyngeal gland of nursing bees. Long-term exposure to high doses of clothianidin (100 ppb) or thiacloprid (8,800) seriously damaged the synthetic machinery for ACh in the hypopharyngeal gland and reduced the content of ACh in brood food substantially by 80%. A comparably high dose of thiacloprid (2,500 ppb) applied acutely did not affect the synthesis of ACh. Thus, chronic exposure and a long time period are required to produce morphological and therewith functional effects on ACh synthesis. Reduced cell size, vacuolization of the secretory cells and regression and fragmentation of the canal cells were observed. The ultrastructural images confirm the loss of the secretory buds. These adverse effects on cellular integrity explain the substantial fall of ACh synthesis. In line with this observation recent papers have described cell necrosis and decreased sizes of the hypopharyngeal glands after exposure of adult honeybees to the neonicotinoid imidacloprid [35, 36].

Moreover, lower doses of clothianidin (1 and 10 ppb) and thiacloprid (200 ppb) reduced ACh content in larval brood. These lower doses can be regarded as being close to field-realistic dosages. Thus, for example maximal thiacloprid residues of 100–199 μg/kg pollen have been detected in apiaries investigated in the USA and Germany [32, 34]. In the present experiments chronic exposure to 200 ppb thiacloprid resulted in a significant reduction of the ACh content in the larval brood. In this context one has to consider that the ACh level of larval brood mirrored the different states of breeding compromisation. Placing hives for 4 weeks in tunnels nearly halved ACh content in brood food on the one hand and reduced brood area and number of capped cells compared to the outside control on the other. Additional exposure to low neonicotinoid doses again nearly halved the ACh content compared to the untreated semi-field control, and the number of capped cells was additionally reduced. Finally, high doses of the neonicotinoids reduced the ACh content by about 80% and breeding success was more or less completely compromised. This correlation, together with the results obtained with the manipulated and ACh-supplemented larval diet, support the significance of non-neuronal ACh for honeybee breeding. Neonicotinoids can compromise larval development of honeybees by reducing the ACh content in the brood food.

Taking all data together, we have shown that the hypopharyngeal glands of nursing honeybees produce high amounts of ACh within an acidic micro-environment, which ensures delivery of active, non-hydrolized ACh directly to the larvae. Thus, the present study delineates an as yet unknown role of non-neuronal ACh during honeybee breeding and possible adverse effects mediated by chronic exposure to neonicotinoids. This mechanism could contribute to the reduced survival of honeybees and should be taken into consideration when reviewing the risk assessment of these compounds.

## Abbreviations

ACh: acetylcholine
ChAT: choline acetyltransferase
HPLC: high pressure liquid chromatography
ppb: parts per billion
RJ: royal jelly
SDS PAGE: sodium dodecyl sulfate polyacrylamide gel electrophoresis
